# Bacterial vitamin B_12_ production enhances nematode predatory behavior

**DOI:** 10.1038/s41396-020-0626-2

**Published:** 2020-03-09

**Authors:** Nermin Akduman, James W. Lightfoot, Waltraud Röseler, Hanh Witte, Wen-Sui Lo, Christian Rödelsperger, Ralf J. Sommer

**Affiliations:** Department for Evolutionary Biology, Max Planck Institute for Developmental Biology, Max Planck Ring 9, 72076 Tübingen, Germany

**Keywords:** Bacterial genetics, Animal physiology

## Abstract

Although the microbiota is known to affect host development, metabolism, and immunity, its impact on host behavior is only beginning to be understood. In order to better characterize behavior modulation by host-associated microorganisms, we investigated how bacteria modulate complex behaviors in the nematode model organism *Pristionchus pacificus*. This nematode is a predator that feeds on the larvae of other nematodes, including *Caenorhabditis elegans*. By growing *P*. *pacificus* on different bacteria and testing their ability to kill *C. elegans*, we reveal large differences in killing efficiencies, with a *Novosphingobium* species showing the strongest enhancement. This enhanced killing was not accompanied by an increase in feeding, which is a phenomenon known as surplus killing, whereby predators kill more prey than necessary for sustenance. Our RNA-seq data demonstrate widespread metabolic rewiring upon exposure to *Novosphingobium*, which facilitated screening of bacterial mutants with altered transcriptional responses. We identified bacterial production of vitamin B_12_ as an important cause of such enhanced predatory behavior. Although vitamin B_12_ is an essential cofactor for detoxification and metabolite biosynthesis, shown previously to accelerate development in *C. elegans*, supplementation with this enzyme cofactor amplified surplus killing in *P. pacificus*, whereas mutants in vitamin B_12_-dependent pathways reduced surplus killing. By demonstrating that production of vitamin B_12_ by host-associated microbiota can affect complex host behaviors, we reveal new connections between animal diet, microbiota, and nervous system.

## Introduction

Organisms harbor and interact with diverse microbial communities depending on their own ecology and environment. Furthermore, the microbiota are considered a fundamental aspect of a host’s biology and are known to provide developmental cues, effect metabolism, and alter immunity [1–3], However, the microbiota constitutes a complex network of microorganisms and disentangling specific interactions at a mechanistic level is challenging. Bacterial-feeding nematodes therefore provide a highly attractive system to study the influence of the microbiota as the specific interactions can be investigated in monoxenic cultures where the microbiota and diet are indistinguishable from one another and easily controlled. Remarkably, despite the abundance of nematode species, often little is known of their ecology and the bacterial associations found naturally between these organisms. For instance, the microbiota of the model nematode *Caenorhabditis elegans* was only recently characterized [4–6] and shown to influence host fitness and response to pathogens [5, 7]. Furthermore, its microbiota is thought capable of synthesizing all the essential nutrients *C. elegans* may require [8].

**Fig. 1:**
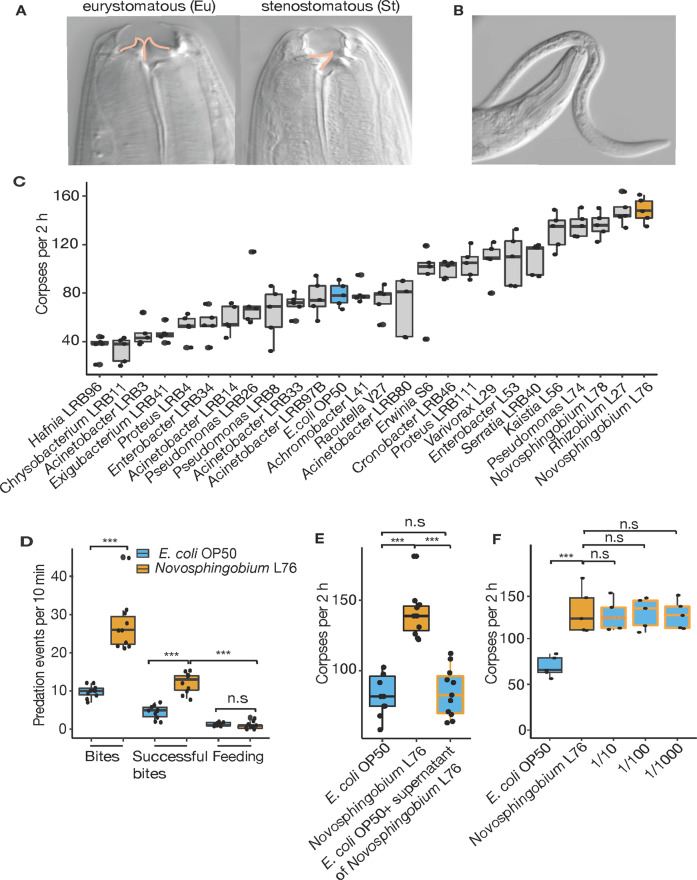
Bacterial diet modulates killing behavior in *P. pacificus*. **a** Eurystomatous (Eu) and stenostomatous (St) mouth forms. Eu worms are capable of predation and have a wide mouth with two teeth, whereas St worms feed on bacteria and have a narrower mouth with one tooth. **b** A predatory *P*. *pacificus* adult biting a *C*. *elegans* larvae. **c** Corpse assay of *P*. *pacificus* predators fed upon *C*. *elegans* larvae following growth on a variety of bacteria from *Pristionchus*-associated environments; five predators are fed prey for 2 h for each assay. *N* = 5 replicates for each assay. **d** Bite assay after growth on either an *E*. *coli* OP50 or *Novosphingobium* L76 diet to assess the effect on *P*. *pacificus* surplus-killing behavior. Numbers of bites, successful bites and feeding was quantified during a 10 min interval while fed upon *C*. *elegans* larvae. **e** Corpse assay of *P*. *pacificus* fed with *E*. *coli* OP50, *Novosphingobium* L76 or of *E*. *coli* OP50 with *Novosphingobium* L76 supernatant. *N* = 10 replicates for each assay for **d** and **e**. **f** Corpse assays of *P*. *pacificus* previously fed with a mixture of *Novosphingobium* L76 and *E*. *coli* OP50 at 1/10, 1/100, and 1/1000 concentrations. Low concentrations of *Novosphingobium* L76 in the diet is sufficient to influence killing behavior. Bacteria were spotted to NGM without peptone to prevent bacterial growth. *N* = 10 replicates for each assay.

Another well-characterized nematode species in addition to *C. elegans* is *Pristionchus pacificus*, which shows novel ecological, morphological, and behavioral traits not observed in *C. elegans*. *P. pacificus* is a soil nematode frequently found associated with scarab beetles upon which it shares a necromenic association [9]. Here, *P. pacificus* exploits the distinct microbial habitat found on the decaying beetle carcass to complete its life cycle [10]. In addition, *P. pacificus* is an omnivorous nematode capable of feeding on bacteria, fungi, and also predating on other nematodes [11–13]. Predation is dependent on morphological and behavioral novelties, involving the formation of teeth-like denticles and a self-recognition mechanism [14–17]. The ability to form teeth-like denticles is an example of developmental plasticity with two discrete mouth forms [18]. The stenostomatous morph has a single blunt tooth, whereas the eurystomatous morph has two large teeth with only the latter capable of predation (Fig. 1a, b) [12, 14]. Predation may confer a selective advantage in certain environmental settings with previous studies indicating that different culture conditions, including microbial diet, are able to modulate the ratio of the two mouth forms [19, 20]. Furthermore, *P. pacificus* predation under laboratory conditions is also an example of a phenomenon known as surplus-killing behavior [12]. Surplus killing is a well-documented complex behavior observed in many predators across the animal kingdom, in which more prey are killed than nutritionally required [21–27]. Theoretical and experimental studies considered surplus killing a potentially context-dependent, adaptive foraging strategy or alternatively, a context-general syndrome of high aggression [21, 22, 26]. However, the full impact of diet on predation is currently poorly understood.

Here, utilizing previously isolated bacteria from soil, scarab beetles, and figs that are found naturally associated with *Pristionchus* nematodes [28], we investigate their influence on its predatory behavior and predatory associated traits. We analyze 25 different bacterial species and establish their ability to modulate the predatory feeding behaviors including surplus killing. Subsequently, by focusing on one bacterial species in which surplus killing is strongly enhanced, *Novosphingobium*, we conduct a mutant screen and identify bacterial derived vitamin B_12_ as a major component involved in enhancing the predatory behaviors. Furthermore, we demonstrate that the addition of exogenous vitamin B_12_ is sufficient to recapitulate the heightened predatory behaviors, whereas mutations in B_12_-dependent pathways had an opposing effect. Therefore, ecologically relevant bacteria found naturally associated with *P. pacificus* influence behavioral traits.

## Materials and methods

### Nematode and bacterial strains

A list of all nematode and bacterial species and strains can be found in Supplementary Table 1.

### Bacterial culture conditions

All bacterial strains and mutants were grown overnight in LB (Lysogeny broth) supplemented with 50 μg/ml kanamycin where required. Bacteria were grown at 30 °C or 37 °C depending on the species and 6 cm nematode growth medium (NGM) plates were seeded with 50 μl bacterial overnight cultures and were incubated for 2 days.

### Nematode culture conditions

*P. pacificus*, *C. elegans*, *Rhabditophanes* sp. KR302, and *A. sudhausi* were grown under standard nematode growth conditions on NGM plates seeded with *Escherichia coli* OP50. Egg cultures were obtained by treating healthy gravid adults with alkaline hypochlorite (bleaching) and were maintained and raised at 20 °C on NGM plates. The free-living generation of *Parastrongyloides trichosuri* was cultured as described in Grant et al. [29]. Briefly, to maintain the *P. trichosuri* free-living generation in culture, *E. coli* OP50-spotted NGM plates were incubated for 2 days at room temperature (RT). Autoclaved rabbit feces were lightly broken and placed on the spotted NGM plate along with *P. trichosuri* animals. Additional *E. coli* OP50 (supplemented with/without vitamin B_12_) was subsequently added to the dry rabbit feces. The entomopathogenic nematode *Steinernema carpocapsae* was grown on its symbiotic bacterium *Xenorhabdus nematophila*. Symbiotic bacteria were inoculated in LB and incubated at 25 °C overnight, 300 μl from overnight cultures were spotted to NGM plates (supplemented with/without vitamin B12) and incubated for 1 day at RT. *S. carpocapsae* nematodes were transferred to their respective symbiotic bacterial plates and subsequently grown at 20 °C.

### Mouth-form phenotyping

Mouth-form phenotyping was performed as previously reported [12, 13]. In brief, axenic worm eggs were obtained by treating healthy gravid *P. pacificus* adults with alkaline hypochlorite, which were subsequently maintained on the test bacteria strains or mutants for at least two generations. Synchronized J4 stage juvenile larvae were picked onto NGM plates with the same test bacteria and roughly 12 h later, worms became young adults. NGM plates with synchronized young adults were placed onto a stereomicroscope with high magnification (×150). The eurystomatous (Eu) mouth form was determined by the presence of a wide mouth, whereas the stenostomatous (St) forms were determined by a narrow mouth. Eu young adult worms were picked for predation assays.

### Predation assays

We used two types of predation assays as described below.

#### Corpse assays

Corpse assays facilitated rapid quantification of predatory behavior and were conducted as previously described [12, 13, 17]. Briefly, in order to generate substantial *C. elegans* larvae for use as prey, cultures were maintained on *E. coli* OP50 bacteria until freshly starved resulting in an abundance of young larvae. These plates were washed with M9 buffer, passed through two Millipore 20 μm filters and centrifuged at 377 × *g* to form a concentrated larval pellet of juvenile animals. Excess buffer was removed and 1 μl of worm pellet was deposited onto a 6 cm NGM unseeded assay plates. This resulted in roughly 3000 prey larvae on each assay plate. Assay plates were left for a minimum of 1 h to allow larvae to distribute evenly over the plate. Young adult *P. pacificus* predators were screened for the predatory Eu mouth form and transferred to empty NGM plates for 30 min to remove any excess bacteria from their bodies. Subsequently, five *P. pacificus* nematodes were added to each assay plate. Predators were permitted to feed on the prey for 2 h before removal and the plate was subsequently screened for the presence of larval corpses, which were identified by the absence of motility coinciding with obvious morphological defects including leaking innards or missing worm fragments. Each assay was replicated ≥5 times. When post-feeding size measurement was required, predatory animals were picked to NGM plates containing no bacteria and measurements were taken using the Wormsizer plug in for Image J/Fiji [30]. See below for Wormsizer experimental details.

#### Bite assays

Bite assays provide a more detailed and thorough analysis of the specific interactions associated with predatory behaviors. Bite assays were conducted as previously described [12, 13, 17]. Briefly, substantial *C. elegans* prey was generated by maintaining *C. elegans* cultures on *E. coli* OP50 bacteria until freshly starved resulting in an abundance of young larvae. These plates were washed with M9 buffer, passed through two Millipore 20 μm filters and centrifuged at 377 × *g* to form a concentrated larval pellet of juvenile animals. Excess buffer was removed and 1 μl of worm pellet was deposited onto a 6 cm NGM unseeded assay plate. This resulted in roughly 3000 prey larvae on each assay plate. Assay plates were left for a minimum of 1 h to allow larvae to distribute evenly over the plate. Young adult *P. pacificus* predators were screened for the appropriate predatory Eu mouth morph and transferred to empty NGM plates for 30 min to remove any excess bacteria from their bodies. A single predator was placed on to the assay plate and allowed to recover for 20 min. After recovery, the predatory animal was directly observed under a light stereomicroscope for 10 min and the number of bites, successful bites and feeding events quantified. “Bites” were characterized by a switch to the slower predatory pharyngeal pumping rhythms previously described [12, 13, 17] coinciding with a restriction in movement of the prey. “Successful bites” were characterized by successful rupturing of the prey cuticle resulting in sufficient damage to cause the innards to leak from the wound. “Feeding” was characterized by consumption of the prey through either the observation of prolonged predatory feeding rhythms once the predator had successful grasped its prey, or alternatively, observation of the faster bacterial associated feeding rhythms at the site of a puncture wound. In these assays, no distinction was made as to whether the predatory behavior events were against live prey or against recently killed or wounded animals. Indeed, predators were occasionally observed repeatedly biting the same dying or dead larvae and each contact was quantified as a distinct predatory event. Each assay was conducted with ten different animals.

### Pharyngeal pumping analysis

*P. pacificus* worms were maintained on 6 cm NGM agar plates and fed on the appropriate test bacterial strains prior to assaying. Young adults were transferred onto assay plates and allowed to recover for 15 min from the stress of being transferred. Worms were observed on a Zeiss microscope at ×40–×63 magnifications, with a high-speed camera and pharyngeal pumping was recorded for 15 s, at 50 Hz in at least 20 animals to ensure accurate quantification. The recorded movies were replayed at the desired speed to count individual pumps as previously described [6].

### *E. coli* OP50 supplementation with *Novosphingobium* L76 supernatant

*E. coli* OP50 and *Novosphingobium* L76 were grown overnight in LB at 37 °C and 30 °C, respectively. Five milliliters overnight cultures of each bacteria were grown until they measured an OD_600_ 1. Bacterial cultures were centrifuged at 10,000 rpm, RT for 5 min and supernatants were isolated by filtering with 5 μm filters. The *E. coli* OP50 pellet was re-suspended with 5 ml *Novosphingobium* L76 supernatant. Three hundred microliters of the *E. coli* OP50 with *Novosphingobium* L76 supernatant was subsequently spotted to 6 cm NGM plates. OP50 pellet with OP50 supernatant and additionally, *Novosphingobium* L76 were also spotted to 6 cm NGM plates as controls. Spotted NGM plates were ready for assay after 2 days of incubation. Freshly bleached eggs from well-grown *P. pacificus* cultures were then transferred onto assay plates and worms were transferred to new assay plates 2 days later. Worms were grown until young adult stage and synchronized young adults were picked and assessed via corpse assays.

### Mixing bacterial diets

Liquid cultures of *E. coli* OP50 and *Novosphingobium* L76 were grown in LB at 37 °C and 30 °C, respectively. Bacterial cultures were diluted to the same OD_600_ and mixed in ratios 1/10, 1/100, and 1/1000. Bacterial suspensions were spread onto peptone-free NGM plates to minimize bacterial growth and plates were briefly air dried in a sterile hood. Bleached *P. pacificus* eggs were added to the plates and worms were allowed to grow until young adult stage; synchronized young adults were then picked and assessed via corpse assays.

### Switching bacterial diet

Overnight cultures of *E. coli* OP50 and *Novosphingobium* L76 were spread to NGM plates and incubated at RT for 2 days. Subsequently, bleached *P. pacificus* eggs were added to the *E. coli* OP50 plates. Worms were transferred from these *E. coli* OP50 plates to *Novosphingobium* L76 at specific developmental stages, L2, L3, and L4, respectively, and were allowed to develop into young adult stage on *Novosphingobium* L76. Worms fed with *E. coli* OP50 or *Novosphingobium* L76 from egg to young adult stage were used as controls. Synchronized young adults were then picked and assessed via corpse assays.

### RNA sequencing

Bacterial strains were grown in LB overnight and spotted to 6 cm NGM plates. Starting from bleached eggs *P. pacificus* nematodes were grown on bacteria for at least two generations and 50 young adults were picked for RNA isolation. Total RNA was extracted using Direct-Zol RNA Mini prep kit (Zymo Research) according to the manufacturer’s guidelines. RNA libraries were prepared by following Truseq RNA library prep kit according to the manufacturer’s guidelines from 1 μg of total RNA in each sample (Illumina Company). Libraries were quantified using a combination of Qubit and Bioanalyzer (Agilent Technologies) and normalized to 2.5 nM. Samples were subsequently sequenced as 150 bp paired end reads on multiplexed lanes of an Illumina HiSeq3000 (IIlumina Inc). Raw reads have been uploaded to the European Nucleotide archive under the study accession PRJEB33410.

### Analysis of RNA-seq data

The software TopHat (version:2.0.14) was used to align raw reads against the *P. pacificus* reference genome (pristionchus.org, version: Hybrid1) and tests for differential expression were performed by Cuffdiff (version: 2.2.1) [31]. Genes with an FDR-corrected *p* value < 0.05 were considered as significantly differentially expressed. For upregulated and downregulated genes, the most significantly enriched metabolic pathways were identified as described previously [19].

### Generation of transgenic lines

We selected the genes *Ppa-stdh-1* and *Ppa-acs-19.1* to generate transcriptional reporters and established transgenic lines necessary for their use as dietary sensors. For *Ppa-stdh-1*, a 2.3 kb interval encompassing the upstream region and the first two exons was amplified. For *Ppa-acs-19.1*, a 1.4 kb region upstream of the first predicted exon was amplified. These promoters were fused to TurboRFP (Evrogen), together with the 3′ UTR sequence of the gene *Ppa-rpl-23* using the primers listed in Supplementary Table 1.

PCR fragments were assembled using Gibson assembly kit (NEB) and verified by Sanger sequencing. The *Ppa-stdh-1*::RFP and *Ppa-acs-19.1*::RFP constructs were amplified with the addition of restriction sites (XmaI and PstI) for subsequent digestion. To form stable lines via the formation of complex arrays, the expression construct *Ppa-stdh-1*::RFP was digested with PstI and 5 ng/μl of this, co-injected into the germlines of young adult *P. pacificus* worms with the marker *Ppa-egl-20*::Venus (10 ng/μl), and genomic carrier DNA (60 ng/μl), also digested with PstI [32]. For the *Ppa-acs-19.1*::RFP construct, 10 ng/μl of the construct cut with PstI, was injected with the marker *Ppa-egl-20*::RFP (10 ng/μl), and genomic carrier DNA (60 ng/μl) also cut with PstI. At least two independent lines were obtained from microinjections for both transgenes.

### Transposon mutagenesis of bacteria

To generate electro-competent cells of *N. lindaniclasticum* LE124 for electroporation, *N. lindaniclasticum* LE124 cells were grown in LB overnight at 30 °C. These overnight cultures were diluted (1:10 vol/vol) and incubated for ≅6 h to reach early log phase (optical density [OD] at 600 nm of 0.3). The culture was centrifuged at 4 °C, 10,000 rpm for 10 min before being washed once with ice-cold distillated water and two times with ice-cold 10% glycerol. After the final washing step, cells were centrifuged and the pellet re-suspended with ≅1 ml 10% glycerol before 50 μl aliquots were distributed to 1.5 ml Eppendorf tubes. The cells in glycerol were electroporated with the EZ-Tn*5* R6Kγ*ori*/KAN-2>Tnp transposon (Epicentre, Madison WI) using an Eppendorf Electroporator 2510 at 2.5 kV yielding around 5 ms. After electroporation, the sample was immediately mixed with SOC (super optimal broth with catabolite repression) medium and incubated at 30 °C for 2 h, the culture was then plated on LB agar medium supplemented with 50 μg/ml of kanamycin.

### Bacterial transposon mutagenesis library preparation

After 2 days incubation of the bacteria at 30 °C, ten colonies were randomly selected, picked and a PCR carried out together with Sanger sequencing to confirm the integration of the transposon into the *N. lindaniclasticum* LE124 genome using the primers KAN-2 FP-1-F (5′-ACCTACAACAAAGCTCTCATCAACC-3′) and R6KAN-2 RP-1 -R (5′-CTACCCTGTGGAACACCTACATCT-3′). After successful confirmation of the bacterial transposon mutagenesis, around 4500 single mutant colonies were picked and inoculated to 96-well plates in 160 μl LB supplemented with 50 μg/ml of kanamycin. Overnight cultures of all mutants were mixed with 160 μl 50% glycerol and frozen at −80 °C.

### Transposon mutant library screening using dietary sensors

Transposon mutants were inoculated into 96-well plates in 180 μl LB supplemented with 50 μg/ml of kanamycin. After overnight growth at 30 °C, 20 μl from the mutant cultures were spotted to 24-well NGM plates. Bacterial mutant strains were incubated for 2 days and eggs of *P. pacificus* RS3271 (*Ppa-stdh-1::*RFP*)* or *P. pacificus* RS3379 (*Ppa-acs-19.1*::RFP) were bleached and filtered with Millipore 120 μm filters to reduce the amount of adult worm carcasses. Around 50–100 bleached eggs were spotted to each well with mutant bacteria; *E. coli OP50* and *N. lindaniclasticum* LE124 wild-type strain were used as controls. Fluorescent worms were grown on the bacterial strains until they became young adults. The *Ppa-stdh-1::*RFP line was screened for decreased RFP expression, whereas the *Ppa-acs-19.1*::RFP line was screened for increased RFP expression. Initial positive results were re-screened at least three times to confirm changes in gene expression.

### Analysis of transposon mutant sequencing data

Raw reads were aligned against *N. lindaniclasticum* LE124 reference genome and transposon sequence by the BWA aln and samse programs (version 0.7.12-r1039) [33]. The generated sam files were screened for read pairs where one read aligned to the transposon sequence and the second read was unmapped. For each mutant line a single gene harboring a transposon insertion site was identified by realignment of the unmapped second read against the *N. lindaniclasticum* LE124 reference with the help of blastn (version: 2.6.0) [34]. Raw whole-genome sequencing data of these mutant lines is available at the European Nucleotide archive under the study accession PRJEB33410.

### Generation of CRISPR-induced mutants of *Ppa-metr-1* and *Ppa-mce-1*

We generated mutant alleles for *Ppa-metr-1* and *Ppa-mce-1* using the CRISPR/Cas9 technique following the protocol described previously [35]. crRNAs were synthesized by Integrated DNA Technologies and fused to tracrRNA (also Integrated DNA Technologies) at 95 °C for 5 min before the addition of the Cas9 endonuclease (New England Biolab). After a further 5 min incubation at RT, TE buffer was added to a final concentration of 18.1 μM for the sgRNA and 2.5 μM for Cas9. Around 20 young adults were injected; eggs from injected P0s were recovered up to 16 h post injection. After hatching and 2 days of growth these F1 were picked onto individual plates until they had also developed and laid eggs. The genotype of the F1 animals was subsequently analyzed via Sanger sequencing and mutations identified and isolated in homozygosity.

### Phylogenetic analysis

For two fatty acid metabolism related genes with differential expression between the bacterial diets, we retrieved homologs by BLASTP searches against WormBase (version: WS270) and pristionchus.org (version: TAU2011). Homologous protein sequences from *C. elegans* and *P. pacificus* were aligned by MUSCLE (version: 3.8.31) [36] and maximum likelihood trees were generated with the help of the phangorn package in R (version: 3.5.3, parameters: model **=** “LG”, optNni = TRUE, optBf = TRUE, optInv = TRUE) [37]. To assess the robustness of the resulting trees, 100 bootstrap pseudoreplicates were calculated. For two *C. elegans* candidate genes involved in the Vitamin B_12_ pathway, one-to-one orthologs in *P. pacificus* could directly be retrieved from BLASTP searches against WormBase (version: WS270): *Ppa-metr-1* (PPA25255) and *Ppa-mce-1* (PPA39850). One-to-one orthology was confirmed by phylogenetic analysis.

### Metabolite supplementation

Methylcobalamin (Vitamin B_12_ CAS Number 13422–55–4) and l-methionine (CAS Number 63–68–3) were purchased from Sigma and dissolved in water at the highest possible soluble concentrations to prepare stock solution. A methylcobalamin stock was prepared fresh before use in each experiment. Metabolite solutions were mixed with NGM agar at the required concentration just before pouring the 6 cm plates. Plates were allowed to dry at RT for 2 days and then spotted with *E. coli* OP50. We first tested different concentrations of vitamin B_12_ and found the strongest and most reliable effect with a concentration of 500 nM, which is most likely un-physiological. In *C. elegans*, similar dose-dependent effects have been seen for vitamin B_12_ [38].

### *Ppa*-*acs-19.1*::RFP gene expression screening on metabolite-supplemented plates

We used *Ppa*-*acs-19.1*::RFP transgenic animals to determine working concentrations of metabolite supplementations. Bleached *Ppa*-*acs-19.1*::RFP transgenic eggs were transferred to metabolite-supplemented plates, which were prepared as described above. *Ppa*-*acs-19.1*::RFP positive young adults were screened for differences in gene expression in comparison to control animals grown on a *E. coli* OP50 and *N. lindaniclasticum* LE124 diet without metabolite supplementation.

### Imaging transgenic reporter lines

Eggs of transgenic reporter lines *Ppa*-*acs-19.1*::RFP and *Ppa-stdh-1*::RFP were bleached and transferred to bacteria plates that were prepared as described. Three milliliters of 2% agar was prepared and a drop (150 μl) of 1 M sodium azide (NaN_3_) was added and mixed with agar to immobilize the worms. Around 200 μl agar was dropped on microscope slide and young adult transgenic worms were placed on the agar. Images of the worms were taken with 10× objective of ZEISS Imager Z1 equipped with the AxioCam camera using ZEN imaging software. The same exposure time was applied to all images.

### Vitamin B_12_ (Methylcobalamin) supplementation assays

Vitamin B_12_-supplemented plates were prepared as described above. *P. pacificus*, *C. elegans*, *Rhabditophanes* sp. KR3021*, A. sudhausi* SB413, as well as *Ppa-metr-1* (*tu1436, tu1436*) and *Ppa-mce-1*(*tu1433, tu1434* and *tu1435*) mutant animals were grown on supplemented plates from egg to young adult stage and subsequently used for (i) predatory assays, (ii) worm size measurements, and (iii) developmental assays. For supplementation experiments with free-living *P. trichosuri*, juvenile larvae (J2 stage) were washed five times with M9 medium and filtered with Millipore 20 μm filters before being soaked in PBS supplemented with 100 μg/ml penicillin and ampicillin for 1 h to avoid contamination. juvenile larvae (J2 stage) were washed a final time with PBS containing no antibiotics and transferred to assay plates. For *S. carpocapsae*, juvenile larvae (J2 stage) were washed with M9 medium and filtered with Millipore 20 μm filters before transferring to NGM plates supplemented with/without 500 nM vitamin B_12_.

### Worm size measurement

*P. pacificus*, *C. elegans*, *Rhabditophanes* sp., *P. trichosuri, A. sudhausi*, and *S. carpocapsae* synchronized young adults were transferred from assay plates to NGM plates without bacteria. Bright field images of the worms were taken using 0.63× objective of ZEISS SteREO Discovery V12 using the AxioCam camera. Images were analyzed using the Wormsizer plug in for Image J/Fiji [30]. Wormsizer detects and measures the volume of the worms.

### Development rate assays

For development rate assays, *P. pacificus*, *C. elegans, Rhabditophanes sp*., and *A. sudhausi* were grown on OP50 at 20 °C. Nematode eggs were bleached, washed with M9 several times and allowed to hatch in M9 medium for 20 h in the absence of food to cause juvenile arrest at the J2 development stage. Once synchronized, juvenile larvae were filtered through two Millipore 20 μm filters and around 30–60 juvenile (J2) animals were transferred to NGM plates (supplemented with/without 500 nM vitamin B_12_) spotted in 50 μl of the desired test bacterial strain. Nematodes were subsequently allowed to develop on test bacteria for the following time periods: *P. pacificus* 57 h at 20 °C, *C. elegans* and *Rhabditophanes sp*. 45 h at 20 °C and *A. sudhausi* for 144 h at RT. Following this, worms were categorized into groups based on the development of the vulva and germ line using 0.63× objective of ZEISS SteREO Discovery V12 following previously established protocols [38].

### Statistical analysis

Statistical calculations (mean, SEM, and t test) were performed by using R studio software. Pairwise *t*-tests with Benjamini–Hochberg multiple testing correction were applied when testing the effect of a single treatment or mutant against one single control sample. For tests across different groups (e.g., treatments, mutants, behaviors), Tukey-HSD test was applied. Significance is designated between two samples according to the following scale: 0 ‘***’ 0.001 ‘**’ 0.01 ‘*’ 0.05 ‘n.s’ 0.1 ‘n.s’ 1.

## Results

### Bacterial diet modulates complex behaviors in nematodes

We tested the effect of 25 different bacteria recently isolated from *Pristionchus*-associated environments [28] on various predation associated traits (Table S1). Specifically, we grew *P. pacificus* for two generations on monoxenic cultures and investigated the effect on mouth-form ratio, pharyngeal pumping, and killing behavior by comparing them to standard laboratory cultures grown on *Escherichia coli* OP50. While diet had a limited effect on mouth-form ratios, we found up to a fourfold difference in killing efficiency and pharyngeal pumping depending on microbial diet (Figs. 1c and S1A, B). The strongest effect on killing efficiency was observed when *P. pacificus* was fed upon three alpha-proteobacteria of the genera *Novosphingobium* and *Rhizobium*, resulting in up to 160 corpses of dead prey in standardized corpse assays (Fig. 1c). We therefore focused on one bacterium of this group, *Novosphingobium* L76, and its effect on killing efficiency.

Stronger killing efficiency translated into higher rates of surplus killing. Specifically, we performed bite assays to observe individual predators for 10 min to distinguish specific predatory events including biting, successful biting that results in penetration of the cuticle, and feeding on prey larvae (see “Method” section for exact description of terms). When grown on *E. coli* OP50, *P. pacificus* only kills 50% of its prey after biting, and subsequent feeding was only observed in roughly 10% of all cases (Fig. 1d, Movie S1). Using *Novosphingobium* L76, we found that the number of *P. pacificus* bites and successful biting events indeed doubled relative to *E. coli* OP50 grown predators (Fig. 1d). However, we found no increase in feeding on the dead prey (Fig. 1d). Instead, predators rapidly moved over agar plates searching for new prey items. In addition, this behavior required live bacterial food, as worms do not grow on heat killed *Novosphingobium*. Thus, a *Novosphingobium* diet enhances predation and surplus killing.

### A *Novosphingobium* diet alters the expression of *P. pacificus* genes involved in fatty acid metabolism

**Fig. 2:**
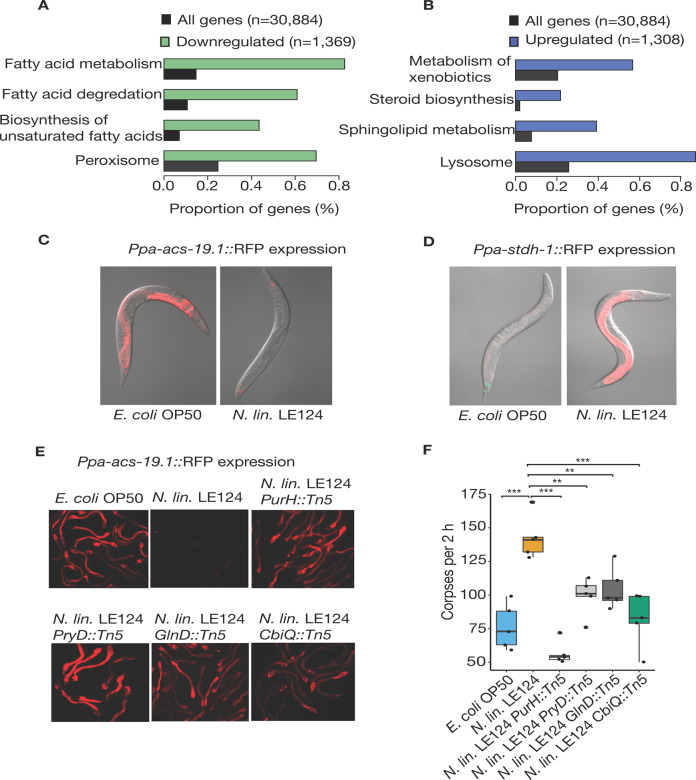
Bacterial diet influences gene expression in *P*. *pacificus*. **a** RNA-seq analysis of *P*. *pacificus* in response to a diet of *Novosphingobium* L76 compared with *E*. *coli* OP50. The pathways with most significant enrichment (FDR-corrected *P* < 0^−5^) in downregulated and (**b**) upregulated genes are shown. **c** The dietary sensor *Ppa-acs-19.1*::RFP is highly expressed in ventral gland, hypodermal and intestinal cells following an *E*. *coli* OP50 diet, whereas a *N*. *lin*. LE124 diet induces expression only in ventral gland cells. The co-injection marker *Ppa-egl-20*::RFP is expressed in the tail. **d** *Ppa-stdh-1:*:RFP is expressed in the intestinal and hypodermal cells with expression strongly upregulated on *N*. *lin*. LE124 compared with an *E*. *coli* OP50 diet. **e** Expression of the *Ppa-acs-19.1*::RFP dietary sensor after feeding on *N*. *lin*. LE124 transposon mutants with mutations in vitamin B_12_ (*N. lin*. LE124 *CbiQ::Tn5)*, purine (*N*. *lin*. LE124 *PurH::Tn5*), pyrimidine biosynthesis (*N*. *lin*. LE124 *PryD::Tn5*) and nitrogen metabolism (*N*. *lin*. LE124 *GlnD::Tn5*). Mutants increase the expression of the dietary sensor in comparison to a *N*. *lin*. LE124 wild-type diet. **f** Corpse assay of *P*. *pacificus* after feeding on various *N*. *lin*. LE124 mutants. There is decreased killing efficiency compared to a *N*. *lin*. LE124 wild-type diet. *N* = 10 replicates for each assay.

Next, we established the necessary bacterial exposure time required to influence predatory behavior and additionally, wanted to know whether the increase in killing was mediated by factors secreted by the bacteria or solely by their ingestion. Only a limited exposure to a diet of *Novosphingobium* L76 during development was sufficient for *P. pacificus* nematodes to exhibit increased predatory behavior (Fig. S1C). In contrast, *Novosphingobium* L76 culture supernatants alone were unable to recapitulate this effect (Fig. 1e). When *Novosphingobium* was diluted with *E. coli* OP50, the effect still persisted suggesting that the response to *Novosphingobium* L76 is unlikely due to differences in caloric intake (Fig. 1f). Instead, the behavioral change is likely a result of physiological alterations caused by the different nutritional composition of *Novosphingobium* L76. Therefore, we analyzed the transcriptomic response of young *P. pacificus* adults grown on *Novosphingobium* in comparison with *E. coli*. We identified a total of 2677 (9%) genes with significant differential expression (FDR-corrected *p* value < 0.05) between the two bacterial diets (Table S2). Most strikingly, more than half of all genes that are predicted to be involved in fatty acid metabolism are significantly differentially expressed between the two diets (Fig. 2a, b).

### A mutant screen identified bacterial derived vitamin B_12_ as a metabolite able to enhance surplus killing

To study the mechanisms by which *Novosphingobium* alters fatty acid metabolism and induces behavioral changes, we used an unbiased bacterial mutagenesis approach. We replaced *Novosphingobium* L76 with *Novosphingobium lindaniclasticum* LE124 (*N. lin*. LE124 thereafter), as the latter can easily be manipulated by transposon mutagenesis, has an available genome [39], and induces similar behavioral effects in *P. pacificus* (Fig. S1D). In addition, to detect any physiological changes in *P. pacificus* caused by mutations in the bacteria, two dietary sensors were generated using *P. pacificus* fatty acid metabolism genes that showed differential expression on different bacteria (Fig. 2a, b) [40]. Specifically, we used homologs of the acyl-CoA synthetase enzyme *Ppa-acs-19.1*, which was upregulated on *E. coli* OP50 and downregulated on *Novosphingobium*, as well as the short-chain dehydrogenase reductase enzyme *Ppa-stdh-1*, which has the opposite expression profile (Fig. 2c, d). Both reporter lines confirmed the differential expression that was detected by RNA-seq (Fig. 2c, d). Subsequently, we used these dietary sensors to screen for bacterial mutants that fail to differentially regulate these genes. From a library of 4320 *N. lin*. LE124 mutants, three affected the expression of *Ppa-stdh-1* and 21 altered the expression of *Ppa-acs-19.1*. Whole-genome sequencing of these bacterial mutants identified transposon insertions in genes corresponding to four biological pathways: purine and pyrimidine metabolism, nitrogen metabolism, and vitamin B_12_ (Fig. 2e; Fig. S2A, key resources table). Importantly, in mutants of all four pathways, the change of transcriptomic response coincided with a reduction in predatory behavior including surplus-killing relative to wild-type *N. lin*. LE124 (Figs. 2f and S2B, C). Thus, the dietary sensor allows the identification of factors regulating complex behavioral traits.

**Fig. 3:**
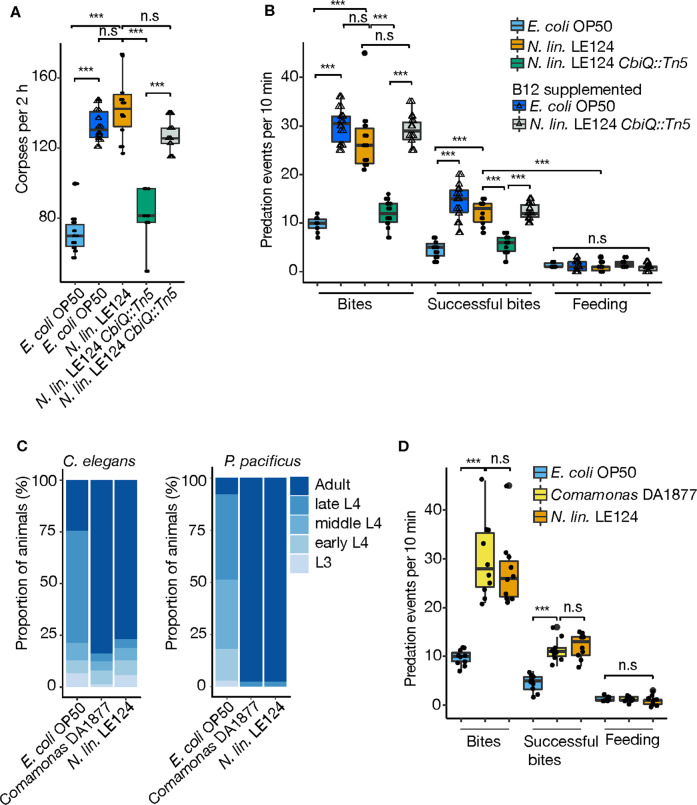
Vitamin B_12_ containing diet regulates surplus-killing behavior and development. **a** Corpse assays showing effects of vitamin B_12_ supplementation on *P*. *pacificus* predation efficiency with *P*. *pacificus* fed on either *E*. *coli* OP50, *N*. *lin*. LE124, *N*. *lin*. LE124 *CbiQ::Tn5*, 500 nM vitamin B_12_ supplemented *E*. *coli* OP50 or 500 nM vitamin B_12_ supplemented *N*. *lin*. LE124 *CbiQ::Tn5* prior to assays. **b** Bite assays showing effects of vitamin B_12_ supplementation on *P*. *pacificus* killing behavior with *P*. *pacificus* fed on either *E*. *coli* OP50, *N*. *lin*. LE124, *N*. *lin*. LE124 *CbiQ::Tn5*, 500 nM vitamin B_12_ supplemented *E. coli* OP50 or 500 nM vitamin B_12_ supplemented *N. lin*. LE124 *CbiQ::Tn5* prior to assays. **c** Developmental staging of *C. elegans* and *P. pacificus* showing percentage of third larval stage (L3), early fourth larval stage (L4), mid fourth larval stage (L4), late fourth larval stage (L4) and young adults on plates after feeding with *E. coli* OP50, *Comamonas* DA18877 and *N. lin*. LE124 for either 45 h (*C. elegans*) or 56 h (*P. pacificus)*. **d** Bite assays of *P. pacificus* fed with *E. coli* OP50, *Comamonas* DA18877 and *N. lin*. LE124. *N* = 10 replicates for each assay in figure.

Vitamin B_12_ has been shown to be a crucial cofactor involved in growth, development and behavior in several animals, including mice and human. Therefore, we focus on vitamin B_12_, which was recently also found to affect growth and development of *C. elegans* [38], whereas nothing is known about vitamin B_12_ affecting *C. elegans* behavior. We first analyzed if vitamin B_12_ supplementation was sufficient to affect the expression of the *Ppa-acs-19.1* sensor and determined the required concentration for this. Supplementation of an *E. coli* diet with 500 nM vitamin B_12_ resulted in the absence of *Ppa-acs-19.1* expression with no adverse effects to the health of wild-type animals (Fig. S3A). In addition, this vitamin B_12_ concentration abolished *Ppa-acs-19.1* expression on *N. lin*. LE124 *CbiQ::Tn5* mutants (Fig. S3B). Subsequently, we analyzed if this supplementation was also sufficient to enhance the predatory behaviors. Indeed, supplementation with 500 nM vitamin B_12_ rescued the vitamin B_12_-deficient *N. lin*. LE124 *CbiQ* mutant and similarly, increased surplus-killing behavior on an *E. coli* diet (Fig. 3a, b). These results demonstrate that vitamin B_12_ is an important micronutrient involved in complex behaviors in nematodes.

### Vitamin B_12_ affects *P. pacificus* development and growth similar to *C. elegans*

Studies by Walhout and co-workers in *C. elegans* showed that developmental acceleration under a *Comamonas aq*. DA1877 diet was also due to vitamin B_12_ [31]. Given the similarities of the *C. elegans* developmental response to *Comamonas* DA1877 and the behavioral response of *P. pacificus* to *N. lin*. LE124, we compared the effect of both bacteria on development and behavior. Indeed, *Comamonas* DA1877 as well as *N. lin*. LE124 induced developmental acceleration of *C. elegans* and *P. pacificus* (Fig. 3c). Similarly, both bacteria enhanced predatory behaviors of *P. pacificus* (Fig. 3d). Thus, the differential effect of bacterial diet on nematode development and behavior might often be due to the uneven distribution of vitamin B12 biosynthesis capabilities of bacteria, but this remains to be tested.

### Two vitamin B_12_-dependent pathways are required for enhanced surplus killing

**Fig. 4:**
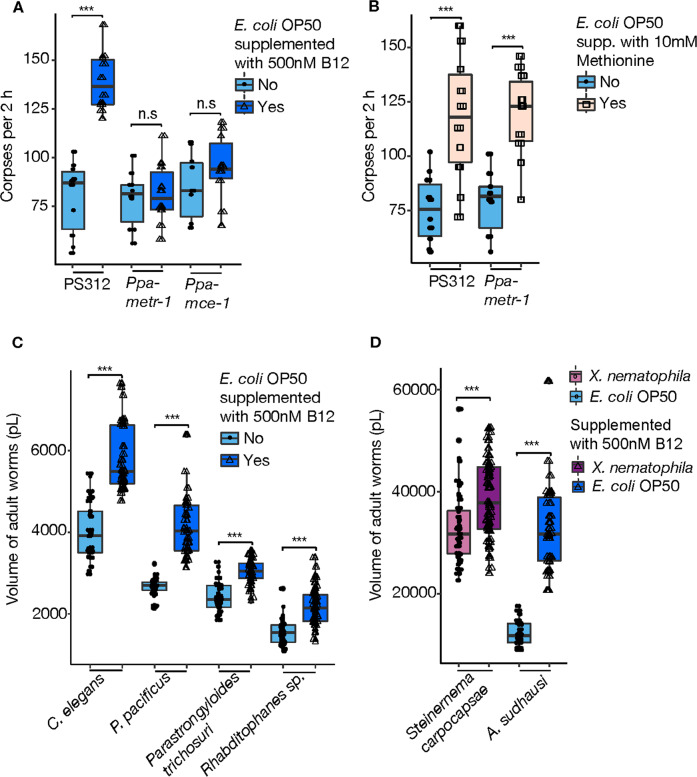
Vitamin B_12_ influence on development is conserved in various nematodes. **a** Corpse assays of *P. pacificus* wild-type (PS312) and mutant animals defective in vitamin B_12_-dependent pathways *Ppa-metr-1* and *Ppa-mce-1* fed with *E. coli* OP50 supplemented with/without 500 nM vitamin B_12_. **b** Corpse assays of PS312 and *Ppa-metr-1* fed with *E. coli* OP50 supplemented with/without 10 mM methionine. *N* = 10 replicates for each assay. **c**, **d** Comparative volume measurement of *C. elegans*, *P. pacificus*, *Parastrongyloides trichosuri*, *Rhabditophanes* sp., *Steinernema carpocapsae and Allodiplogaster sudhausi* after growing on bacterial plates supplemented or not-supplemented with vitamin B_12_. *N* = 60 for each assay. The entomopathogenic nematode *Steinernema carpocapsae* can only be cultured using the specific symbiontic bacterium *Xenorhabdus nematophila*.

In many animals and humans, vitamin B_12_ is a cofactor for two enzymes in different pathways (Fig. S4A). Methionine-synthase (MS) converts homocysteine to methionine in the cytosolic methionine/S-adenosylmethionine (SAM) cycle and in *C. elegans* is encoded by the *metr-1* gene. The second enzyme, methylmalonyl coenzyme A (CoA) mutase, converts methylmalonyl-CoA to succinyl-CoA in mitochondria and is encoded by the *mce-1* gene in *C. elegans*. In humans, vitamin B_12_ deficiency causes methylmalonic aciduria and homocysteinemia resulting in devastating diseases. To test if both pathways are required for increased killing behavior in *P. pacificus*, we generated CRISPR/Cas9-derived mutants in *Ppa-metr-1* and *Ppa-mce-1* (Fig. S4B, C, D). Both mutants failed to respond to the supplementation of an *E. coli* diet with vitamin B_12_ (Fig. 4a). Given that SAM is a donor of methyl-groups for many different substrates including RNA, DNA, and proteins, we supplemented an *E. coli* diet of *P. pacificus* wild type and *Ppa-metr-1* mutant animals with methionine. In both cases, methionine supplementation resulted in enhanced killing behavior when predators were fed *E. coli* bacteria (Fig. 4b). Thus, both vitamin B_12_-dependent pathways seem to be involved in *P. pacificus* predatory behaviors.

### Vitamin B_12_ dependent developmental acceleration is conserved across nematodes

The experiments described above indicate crucial roles of bacterial derived vitamin B_12_ for the development and behavior of both *P. pacificus* and *C. elegans*. As these nematodes are estimated to have diverged roughly 100 Mya [41], we next tested how prevalent the effects of vitamin B_12_ are on the development and physiology of other nematodes, including more distantly related species and representatives that live in diverse ecological settings (Supplementary Table 1). We grew six nematode species of four major taxonomic clades on a vitamin B_12_ supplemented diet and measured the effects on their development and growth by quantifying the total worm volume of young adults. In all species tested, we found a significant increase in worm volume (Fig. 4c, d). This included the facultative parasite *Parastrongyloides trichosuri* and the entomopathogenic nematode *Steinernema carpocapsae*. We found the strongest effect on the large free-living nematode *Allodiplogaster sudhausi* that nearly doubled its volume on a vitamin B_12_ supplemented diet (Fig. 4d). Where possible, we also investigated the effects on developmental speed. Similar to the increase in body size, vitamin B_12_ supplementation accelerated the development of *Rhabditophanes* and *Allodiplogaster* (Fig. S4E, F). Taken together, these results demonstrate important physiological and developmental functions of vitamin B_12_ that are shared across many nematode species.

## Discussion

Here, we identified a novel role for nematode-associated microbiota in modulating the complex behavioral trait of predation and therefore, demonstrates a connection between the microbial diet and the nervous system in nematodes. Diverse bacterial species, which have previously been found naturally associated with *Pristionchus* nematodes [28], elicit different effects on the predatory behavioral state after feeding. Some adversely influence predation, whereas others enhance the predatory behaviors. The greatest enhancement in predatory behaviors was observed when *P. pacificus* was fed upon *Novosphingobium* with the increase in killing influenced by bacterial derived vitamin B_12_. In addition, we have revealed a more general, conserved role for vitamin B_12_ in nematode development and growth. Previous studies have shown vitamin B_12_ to be essential for *C. elegans* development with infertility, growth retardation, and a reduction in life-span observed in animals deficient in vitamin B_12_ [38, 42, 43]. In contrast, behavioral effects have not been reported and similarly, mechanisms of vitamin B_12_ deficiency in humans that result in neuropathies are currently unknown. It is important to note that the modulation of predation and surplus killing in *P. pacificus* requires both vitamin B_12_-dependent pathways. Therefore, we speculate that the influence of vitamin B_12_ on these behaviors is multifactorial and might well involve several factors. Specifically, the SAM pathway feeds into the methylation of DNA, RNA, and proteins, but also lipids and neurotransmitters (Fig. S4a). Indeed, both the purine and pyrimidine synthesis pathways were also isolated in our bacterial mutant screen with mutants negatively influencing the predatory behaviors and both are biochemically related to SAM. Thus, the presence of vitamin B_12_ might act through multiple downstream factors, but how it stimulates these effects has yet to be discovered. Most importantly, the neuronal circuits that are directly or indirectly affected by vitamin B_12_ have to be identified in future research. Notably, several neural circuits and neurotransmitter systems of *P. pacificus* have been determined and investigated in detail [12, 13, 44, 45]. Therefore, future studies can reveal the cellular and molecular foci of vitamin B_12_-dependence and the influence of the microbiota on nematode predation.

This study complements previous work [10], which explored the succession and dynamics of the nematode-microbiota environment associated with the decaying beetle carcass on which *Pristionchus* nematodes are frequently found. Whereas our previous omics approach identified the larger scale ecological communities and their changing composition, by focusing on individual bacterial species we have begun to discover the potential complex interactions influencing these environments. However, much of the influence of the microbiota-nematode interactions within this community still remains to be elucidated. This includes how the complex microbial community contributes to the nematode life cycle and how the abundance and composition of the microbiota may drive the dispersal of the nematodes in order to seek new beetle hosts on which to colonize.

## Supplementary information

Suppl Material

Table S2

Movie S1 Surplus killing

## Data Availability

RNA-seq data has been deposited at the European Nucleotide Archive under the study accession PRJEB33410. All other data is available in the main text or the supplementary materials.
