# Correction: Bacterial vitamin B_12_ production enhances nematode predatory behavior

**DOI:** 10.1038/s41396-020-0644-0

**Published:** 2020-04-03

**Authors:** Nermin Akduman, James W. Lightfoot, Waltraud Röseler, Hanh Witte, Wen-Sui Lo, Christian Rödelsperger, Ralf J. Sommer

**Affiliations:** Department for Evolutionary Biology, Max Planck Institute for Developmental Biology, Max Planck Ring 9, 72076 Tübingen, Germany

**Keywords:** Bacterial genetics, Animal physiology

Correction to: *ISMEJ*

10.1038/s41396-020-0626-2

This article was originally published with an erroneous copyright line. The copyright line read “© The Author(s), under exclusive licence to International Society for Microbial Ecology 2020. This article is published with open access” and has now been corrected to “© The Author(s). This article is published with open access” in both PDF and HTML versions of this paper. The publishers wish to apologize for any inconvenience caused.

