# Supplementary material for: Bacterial vitamin B12 production enhances nematode predatory behavior
Source: ISME J. 2020 Mar 9;14(6):1494–507. doi: 10.1038/s41396-020-0626-2 (PMC7242318; doi:10.1038/s41396-020-0626-2)
Supplement: Supplementary file 1 — Suppl Material [file 41396_2020_626_MOESM1_ESM.docx]

## **Supplementary Material for**

**Bacterial vitamin B_12_ production enhances nematode predatory behavior**

**Authors:** **Nermin Akduman^1^, James W. Lightfoot^1^, Waltraud Röseler^1^, Hanh Witte^1^, Wen-Sui Lo^1^, Christian Rödelsperger^1^, Ralf J. Sommer^1*^**

*corresponding author: ralf.sommer@tuebingen.mpg.de

**Affiliations:** ^1^Department for Evolutionary Biology, Max Planck Institute for Developmental Biology, Max Planck Ring 9, 72076 Tübingen Germany

**Suppelemntary Figure Legends**

**Figure S1. Bacterial diet affects predatory behavior in *P. pacificus.***

(**A**) Mouth form ratio of *P. pacificus* PS312 after feeding with 25 different bacteria strains. Bacterial diet fails to influence mouth-form ratio. N=3 replicates for each assay. (**B**) Pharyngeal pumping behavior of *P. pacificus* PS312 on 25 different bacterial diets. N=20 replicates for each assay. (**C**) Corpse assay illustrating effect of bacterial diet switching from *E. coli* OP50 to *Novosphingobium* L76 at particular *P. pacificus* development stages (second, third and fourth larval stages (L2, L3, L4), respectively). Corpse assays were performed with young adults suggesting feeding with *Novosphingobium* L76 at diverse developmental stages modify killing behavior*.* (**D**) Corpse assays of *P. pacificus* previously fed on either *Novosphingobium* L76 or *Novosphingobium* LE124. The increased killing behaviors are observed in both strains of Novosphingobium. N=10 replicates for each assay.

**Figure S2. Mutations in multiple pathways affect dietary sensor expression and predatory behavior.** (**A**) Images of *Ppa-acs-19.1*::RFP dietary sensor showing purine (*N. lin.* LE124 *PurA::Tn5, N. lin.* LE124 *PurD::Tn5, N. lin.* LE124 *PurE::Tn5, N. lin.* LE124 *GuaB::Tn5* and *N. lin.* LE124 *PurM::Tn5)* and pyrimidine biosynthesis (*N. lin.* LE124 *PryE::Tn5*) mutants increase the expression of the dietary sensor in comparison to *N. lin.* LE124 wild-type diet. (**B**) Corpse assays of *P. pacificus* fed with *N. lin.* LE124 mutants from vitamin B_12_ (green), purine (white), pyrimidine biosynthesis (grey) and nitrogen metabolism (dark grey) all decreasing killing efficiency in comparison to *N. lin.* LE124 wild-type diet. N=10 replicates for each assay. (**C**) Bite assays of *P. pacificus* previously fed on *E. coli* OP50*, N. lin.* LE124 and *N. lin.* LE124 mutants from vitamin B_12_ (green), purine (white), pyrimidine biosynthesis (grey) and nitrogen metabolism (dark grey) modulating killing efficiency. Ten replicates for each assay.

**Fig. S3. Vitamin B_12_ regulates fatty acid gene expression and development.**

(**A**) *Ppa-acs-19.1* transgenic worms were grown on NGM plates supplemented with various concentrations of vitamin B_12_. NGM plates without vitamin B_12_ spotted with *E. coli* OP50 and *N. lin.* LE124 were used as controls. Images of transgenic animals were taken to determine the most efficient vitamin B_12_ concentration. Vitamin B_12_ supplemented *E. coli* OP50 phenocopies *N. lin.* LE124 effect on *Ppa-acs-19.1* expression. (**B**) *Ppa-acs-19.1* transgenic worms were added to NGM plates with *N. lin.* LE124 transposon mutants and with/without supplementation with 500 nM vitamin B_12_. *E. coli* OP50 and *N. lin.* LE124 were as controls. Vitamin B_12_ supplementation rescued *Ppa-acs-19.1* expression on *N. lin.* LE124 *CbiQ::Tn5* mutant (blue highlighted box).

**Figure S4. Vitamin B_12_ dependent metabolic pathways**

**(A)** Network of the main two vitamin B_12_-dependent pathways. *P. pacificus* Orthologous of genes labeled in green were mutated with CRISPR/Cas9. Orthologous of red-labeled *acs-19* used as dietary sensor. **(B)** One-to-one orthologs could be identified for *metr-1* **(C)** and *mce-1*. Nodes with bootstrap support ≥ 90/100 are labeled with stars and arrows mark *P. pacificus* genes that were used for functional studies. **(D)** Mutations were induced in both *Ppa-metr-1* and *Ppa-mce-1* using CRISPR/Cas9 with the target locations indicated in both genes (scissors). Mutations induced via CRIPSR/Cas9 are also shown. (**E**) and (**F**) Developmental staging of *Rhabditophanes sp*. and *A. sudhausi* on *E. coli* OP50 NGM plates supplemented with/without vitamin B_12_. The development of *Rhabditophanes sp*. and *A. sudhausi* was accelerated with vitamin B_12_ supplementation. N=10 replicates for each assay.


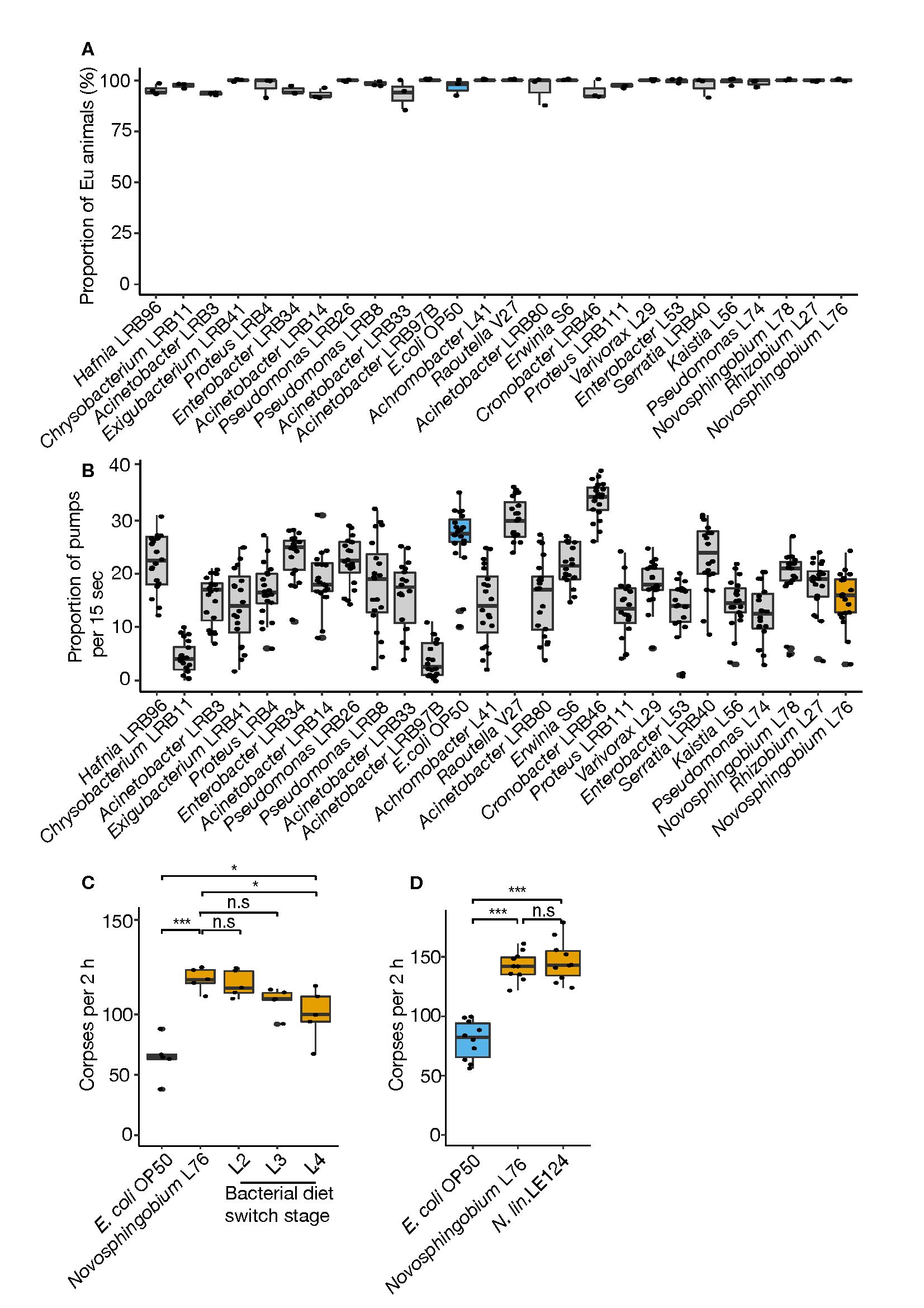


Fig S1

Fig S2

Fig S3


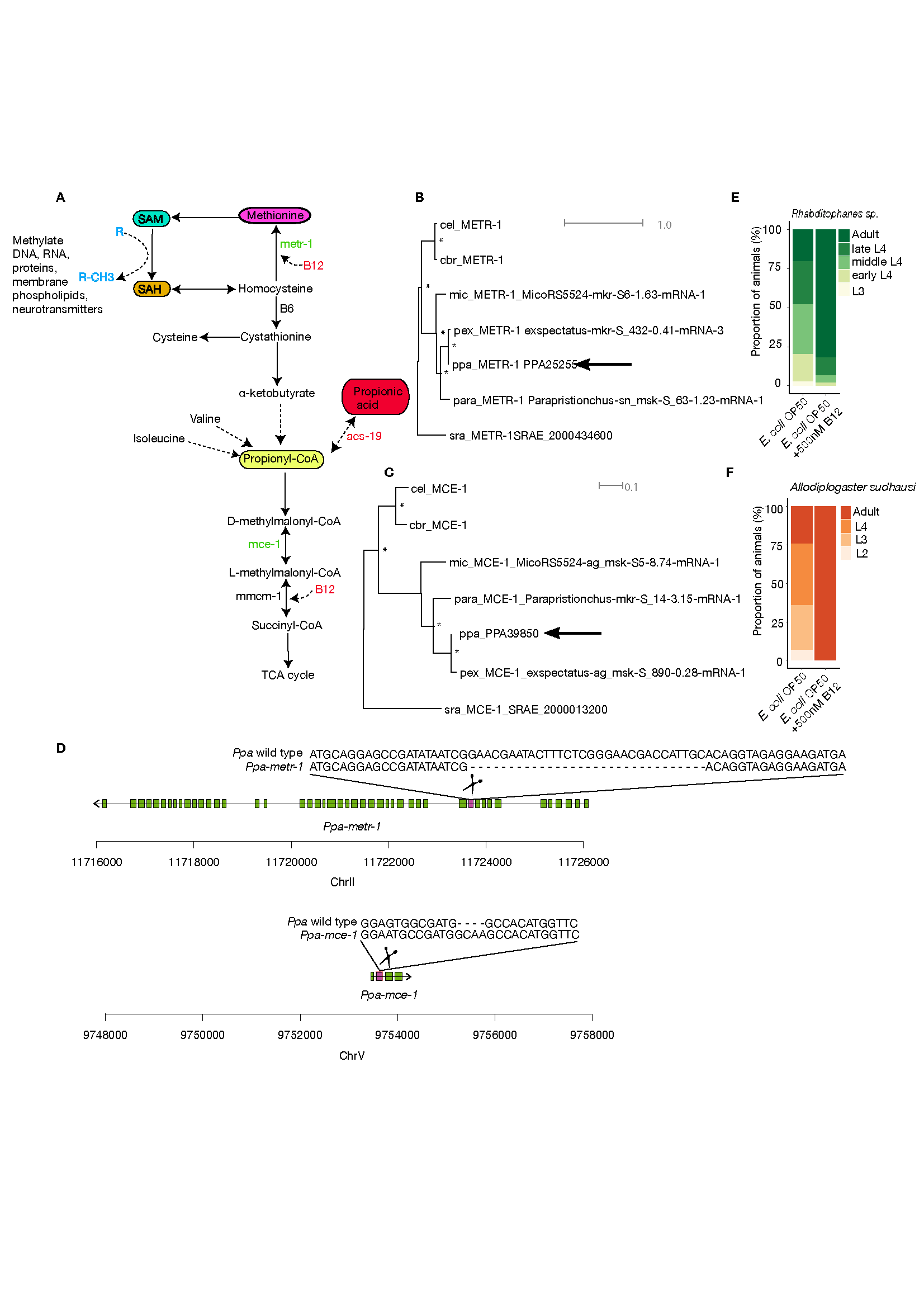


Fig S4

**Supplementary Table 1**

| **REAGENT or RESOURCE** | **SOURCE** | **IDENTIFIER** |
| --- | --- | --- |
| **Bacterial strains** | | |
| *E.coli* OP50 | Caenorhabditis Genetics Center (CGC) | RRID:WB-STRAIN:OP50 |
| *Comamonas aq.* DA1877 | Caenorhabditis Genetics Center (CGC) | N/A |
| *Novosphingobium lindaniclasticum* LE124 | German collection of microorganisms and cell culture GmbH (DSMZ) | N/A |
| *Hafnia LRB96* | Akduman et al., 2018 | N/A |
| *Chrysobacterium* LRB11 | Akduman et al., 2018 | N/A |
| *Acinetobacter* LRB3 | Akduman et al., 2018 | N/A |
| *Exiguobacterium* LRB41 | Akduman et al., 2018 | N/A |
| *Proteus* LRB4 | Akduman et al., 2018 | N/A |
| *Enterobacter* LRB34 | Akduman et al., 2018 | N/A |
| *Acinetobacter* LRB14 | Akduman et al., 2018 | N/A |
| *Pseudomonas* LRB26 | Akduman et al., 2018 | N/A |
| *Pseudomonas* LRB8 | Akduman et al., 2018 | N/A |
| *Acinetobacter* LRB33 | Akduman et al., 2018 | N/A |
| *Acinetobacter* LRB97B | Akduman et al., 2018 | N/A |
| *Achromobacter* L41 | Akduman et al., 2018 | N/A |
| *Raoultella* V27 | Akduman et al., 2018 | N/A |
| *Acinetobacter* LRB80 | Akduman et al., 2018 | N/A |
| *Erwinia* S6 | Akduman et al., 2018 | N/A |
| *Cronobacter* LRB46 | Akduman et al., 2018 | N/A |
| *Proteus* LRB111 | Akduman et al., 2018 | N/A |
| *Variovorax* L29 | Akduman et al., 2018 | N/A |
| *Enterobacter* L53 | Akduman et al., 2018 | N/A |
| *Serratia* LRB40 | Akduman et al., 2018 | N/A |
| *Kaistia* L56 | Akduman et al., 2018 | N/A |
| *Pseudomonas* L74 | Akduman et al., 2018 | N/A |
| *Novosphingobium* L78 | Akduman et al., 2018 | N/A |
| *Rhizobium* L27 | Akduman et al., 2018 | N/A |
| *Novosphingobium* L76 | Akduman et al., 2018 | N/A |
| *Novosphingobium lindaniclasticum* LE124 *PurA::Tn5* | This study | N/A |
| *Novosphingobium lindaniclasticum* LE124 *PurD::Tn5* | This study | N/A |
| *Novosphingobium lindaniclasticum* LE124 *PurE::Tn5* | This study | N/A |
| *Novosphingobium lindaniclasticum* LE124 *PurH::Tn5* | This study | N/A |
| *Novosphingobium lindaniclasticum* LE124 *PurM::Tn5* | This study | N/A |
| *Novosphingobium lindaniclasticum* LE124 *GuaB::Tn5* | This study | N/A |
| *Novosphingobium lindaniclasticum* LE124 *PryD::Tn5* | This study | N/A |
| *Novosphingobium lindaniclasticum* LE124 *PryE::Tn5* | This study | N/A |
| *Novosphingobium lindaniclasticum* LE124 *GlnD::Tn5* | This study | N/A |
| *Novosphingobium lindaniclasticum* LE124 *CbiQ::Tn5* | This study | N/A |
| **Chemicals, Peptides, and Recombinant Proteins** | | |
| Alt-R CRISPR-Cas9 tracrRNA | \|  \| Integrated DNA Technologies \| \| --- \| --- \| | Cat#1072534 |
| EnGen Cas9 NLS, *S. pyogenes* | New England Biolabs | Cat#M0646M |
| Methylcobalamin | Sigma-Aldrich | CAS Number [63-68-3](https://www.sigmaaldrich.com/catalog/search?term=63-68-3&interface=CAS%20No.&N=0&mode=partialmax&lang=de&region=DE&focus=product) |
| L-Methionine | Sigma-Aldrich | Lot#SLBZ1683 |
| Kanamycin | Sigma-Aldrich | CAS Number [70560-51-9](https://www.sigmaaldrich.com/catalog/search?term=70560-51-9&interface=CAS%20No.&N=0&mode=partialmax&lang=de&region=DE&focus=product) |
| FastDigest PstI | Thermofisher Scientfic | Cat# FD0615 |
| FastDigest BamHI | Thermofisher Scientfic | Cat# FD0054 |
| Gibson Assembly^®^ Cloning Kit | New England Biolabs | Cat# E5510S |
| **Experimental Models: Organisms/Strains** | | |
| *Pristionchus pacificus*: strain PS312 | Stock of Dep. IV, MPI Developmental Biology Tuebingen | N/A |
| *C. elegans;*  strain N2 Bristol | Caenorhabditis Genetics Center (CGC) | N/A |
| *Rhabditophanes sp;* strain KR3021 | Stock of Dep. IV, MPI Developmental Biology Tuebingen | N/A |
| Parastrongyloides *trichosuri* | Stock of Dep. IV, MPI Developmental Biology Tuebingen | N/A |
| *Allodiplogaster sudhausi* : strain SB413 | Stock of Dep. IV, MPI Developmental Biology Tuebingen | N/A |
| *Steinernema carpocapsae* | R. Ehlers | N/A |
| *P. pacificus* strain RS3271 (*Ppa-stdh-1::*RFP + *Ppa*-egl-20::Venus) | This study | N/A |
| *P. pacificus* strain RS3379 (Ppa-*acs-19.1*::RFP + *Ppa*-egl-20::RFP) | This study | N/A |
| *Pristionchus pacificus*: strain RS3653: *mce-1* (tu1433) | This study | N/A |
| *Pristionchus pacificus*: strain RS3654: *mce-1* (tu1434) | This study | N/A |
| *Pristionchus pacificus*: strain RS3655: *mce-1* (tu1435) | This study | N/A |
| *Pristionchus pacificus*: strain RS3656: *met-1*(tu1436) | This study | N/A |
| *Pristionchus pacificus*: strain RS3657: *met-1*(tu1437) | This study | N/A |
| **Oligonucleotides** | | |
| *Ppa-stdh-1* - F: 5ʹ-GCCAAGCTTGCATGCCTGCACATGCTATGGAGCGTAGC-3ʹ | This study | N/A |
| *Ppa-stdh-1* - R: 5ʹ-CTGAAAAAAAAAACCCAAGCTTGGGTCCCGAAGACGACGTTGTAGAC-3ʹ; | This study | N/A |
| *Ppa-acs-19.1* *-*F 5ʹ-GGATCCCGTCGACCTGCAGGCATG-3 | This study | N/A |
| *Ppa-acs-19.1* *-*R 5ʹ-ATGAGCGAGCTGATCAAG-3 | This study | N/A |
| *TurboRFP-*F 5ʹ- TGCATGCCTGCAGGTCGACGGGATCCGCCATCACTATGCATTGCTG-3ʹ | This study | N/A |
| *TurboRFP-*R 5ʹ-TCCTTGATCAGCTCGCTCATCTGAACCAGCAAGGGCGATAG-3 | This study | N/A |
| KAN-2 FP-1 Forward Primer  5′-ACCTACAACAAAGCTCTCATCAACC-3′ | Epicentre, Madison WI | Cat#TSM08KR |
| R6KAN-2 RP-1 Reverse Primer  5′-CTACCCTGTGGAACACCTACATCT-3′ | Epicentre, Madison WI | Cat#TSM08KR |
| sgRNA target sequence: exon 2 of mce-1: CCATGTGGCCATCGCCACTC | This study | N/A |
| sgRNA target sequence: exon 11 of metr-1: AAAATGTATCTGGATGCAGG | This study | N/A |
| **Recombinant DNA** |  |  |
| Plasmid: pUC19-*egl-20p::TurboRFP::rpl-23utr* | [Schlager et al., 2009](https://www.sciencedirect.com/science/article/pii/S2211124718307411?via%3Dihub#bib36) | N/A |
| Plasmid: pUC19-*egl-20p::Venus::rpl-23utr* | Okumura et al., 2017 | N/A |
| Plasmid: pUC19-*acs-19.1p::TurboRFP::rpl-23utr* | This study | N/A |
| Plasmid: pUC19-*stdh-1::TurboRFP::rpl-23utr* | This study | N/A |
| EZ-Tn*5* R6Kγ*ori*/KAN-2>Tnp transposon | Epicentre, Madison WI | Cat#TSM08KR |
| **Software and Algorithms** | | |
| FIJI | Schindelin et al., 2012 | N/A |
| R | http://www.r-project.org/ | N/A |
| TopHat (version:2.0.14) | Trapnell et al. 2012 | N/A |
| Cuffdiff (version: 2.2.1) | Trapnell et al. 2012 | N/A |
| **Other** |  | N/A |
| Total RNA was extracted using Direct-zol RNA Kits | Zymo Research | Cat#R2051 |
| Truseq RNA library prep kit was used to prepare RNA libraries | Illumina Company | Cat#RS-122-2001 |
